# Gut Immunity and Microbiota Dysbiosis Are Associated with Altered Bile Acid Metabolism in LPS-Challenged Piglets

**DOI:** 10.1155/2021/6634821

**Published:** 2021-03-26

**Authors:** Xiao Xiao, Yuanzhi Cheng, Jie Fu, Zeqing Lu, Fengqin Wang, Mingliang Jin, Xin Zong, Yizhen Wang

**Affiliations:** ^1^National Engineering Laboratory of Biological Feed Safety and Pollution Prevention and Control, Zhejiang University, Hangzhou 310058, China; ^2^Key Laboratory of Animal Nutrition and Feed Science in Eastern China, Ministry of Agriculture, Zhejiang University, Hangzhou 310058, China

## Abstract

Bacterial infections are among the major factors that cause stress and intestinal diseases in piglets. Lipopolysaccharide (LPS), a major component of the Gram-negative bacteria outer membrane, is commonly employed for inducing an immune response in normal organisms for convenience. The association between LPS stimulation and gut immunity has been reported. However, the effects of gut immunity on microbial homeostasis and metabolism of host, especially bile acid and lipid metabolism in piglets, remain unclear. Hence, in the current study, we elucidated the effect of gut immunity on microbial balance and host metabolism. Twenty-one-day-old healthy piglets (male) were randomly assigned into the CON and LPS groups. After 4 hours of treatment, related tissues and cecal contents were obtained for further analysis. The obtained results showed that stimulated LPS considerably damaged the morphology of intestinal villi and enhanced the relative expression of proinflammatory cytokines. Besides, LPS partially changed the microbial structure as indicated by *β*-diversity and increased operational taxonomic units (OTUs) related to *Oxalobacter* and *Ileibacterium*. Furthermore, bile acid, a large class of gut microbiota metabolites, was also assessed by many proteins related to the enterohepatic circulation of bile acids. It was also revealed that LPS markedly inhibited the mRNA and protein expression of TGR5 and FXR (bile acid receptors) in the ileum, which expressed negative feedback on bile acid de novo synthesis. Additionally, results indicated upregulated mRNA of genes associated with the production of bile acid in the liver tissues. Moreover, LPS reduced the expression of bile acid transporters in the ileum and liver tissues and further disturbed the normal enterohepatic circulation. Taken together, gut immunity and microbial dysbiosis are associated with altered bile acid metabolism in LPS-challenged piglets, which provided theoretical basis for revealing the potential mechanism of intestinal inflammation in swine and seeking nutrients to resist intestinal damage.

## 1. Introduction

The gastrointestinal tract of neonatal piglets is vulnerable to external stimuli, such as weaning, diarrhea, pathogens infection, and hostile environmental condition during the early life period [1–3]. Gut microbiota in the enteric cavity is important for the protection of the host intestine against damage [4]. The host intestine and gut microbiota remain in a steady-state condition under normal physiological conditions. During dysbiosis and intestinal injury, inflammatory bowel disease (IBD) results in a detrimental change of microbial community structure, the rapid proliferation of pathogenic bacteria, and invasion of epithelial and lamina propria cells by pathogens [5, 6]. Conversely, dysregulation or imbalance of gut microbiota also leads to metabolic syndrome and many other diseases, thus affecting the host health negatively for the long term [7, 8]. The gut microbiota and metabolites considerably contribute to the crosstalk between microbiota and host homeostasis, which leads to their participation in the occurrence and development of cardiovascular diseases, host metabolism, immune responses, and energy expenditure [9–13].

Recently, microbiota-derived metabolites, including the endotoxin LPS, bile acids (BAs), trimethylamine N-oxide (TMAO), indolepropionic acid (IPA), and short-chain fatty acids (SCFAs), have attracted wider scientific attentions [11, 14–16], though SCFAs are the most common and abundant metabolites [17]. However, BAs, important endogenous molecules, have gradually been paid more attention in the field of liver diseases and intestinal inflammation [18–20]. Compared with SCFAs, BAs have been identified to be more relevant with gut microbiota in mice feeding on a high-fat diet (HFD) [21]. Studies have revealed that primary BAs synthesized in the liver can be transformed into secondary BAs by the metabolic activities of enteric anaerobic bacteria [22]. At the terminal ileum, the majority of BAs are reabsorbed by multiple protein complexes, such as ileal bile acid-binding protein (Ibabp) and apical sodium-dependent bile acid transporter (ASBT) into enterocytes [23]. This is followed by their secretion into the portal circulation via the basolateral BA transporters organic solute transporter subunit-*α* (OST-*α*), OST-*β*, and multidrug resistance-associated protein 2 (MRP2). BAs are then taken up by transporters into hepatocytes. The underlined procedures consist of the whole enterohepatic circulation of BA [18, 24]. BA de novo synthesis in the liver depends on the regulatory factors such as FXR and its downstream targets in the ileum and liver [23, 25]. The whole circulation of BAs effectively emulsifies and drives the fat-soluble vitamins or lipid absorption in the intestine [26, 27]. Furthermore, BAs have been reported for their participation in the development of metabolic diseases and maintenance of intestinal homeostasis [28]. However, under microbial dysbiosis, whether bile acid metabolism (biosynthesis, transport, and circulation) participates in the process of gut microbiota in regulating host immune and metabolism is not clear.

Thus, this study is aimed at systematically evaluating the role of intestinal injury in changing the gut microbial composition, disturbing the bile acid metabolism, and disturbing lipid metabolism in a piglet model. The intestinal injury model was conducted by intraperitoneal injection of LPS, and then gut immunity, microbial balance, bile acid, and lipid metabolism were measured. Our results showed that LPS stimulation in piglets resulted in the occurrence of inflammation both in the ileum and liver, and further disturbed microbial homeostasis with alteration of bile acid and lipid metabolism.

## 2. Materials and Methods

### 2.1. Animals and Experimental Treatments

The approval for each animal experiment was provided by the Animal Care and Use Committee of Zhejiang University (Hangzhou, China) and was in accordance with the institutional guidelines. A total of twenty healthy male 21-day-old piglets (Duroc × Landrace × Yorkshire, body weight of 7.05 ± 0.13 kg) were purchased and then randomly assigned into two groups (*n* = 10 per treatment). Piglets were injected intraperitoneally with phosphate-buffered solution (PBS, the equivalent volume to LPS) or LPS (10 mg/kg body weight). Then after 4 h, the piglets were slaughtered, followed by the collection of samples for further evaluation.

### 2.2. Sample Collection and Processing

After 4 h treatment with LPS, piglets were sacrificed. The distal ileum was fixed in 4% paraformaldehyde, while the freezing of the remaining ileum, whole colon, and liver tissues was carried out in liquid nitrogen, followed by their storage at -80°C until analysis. Fresh cecal contents from each individual were collected into sterile plastic tubes and then frozen in liquid nitrogen, followed by storage at -80°C until DNA extraction.

### 2.3. Intestinal Morphology

For distal ileum histological analyses, formalin-fixed and paraffin-embedded tissues were cut into thick (4 *μ*m) sections, followed by slicing as well as staining with haematoxylin and eosin (H&E). Leica DM3000 Microsystem was used for obtaining the images of the slices. Next, the height of the villi and the depth of the crypt were evaluated by Image-Pro Plus software (IPP; produced by Media Cybernetics Corporation, USA).

### 2.4. RNA Extraction and Real-Time Quantitative PCR (q-PCR) Analysis

TRIzol reagent (Invitrogen, USA) was used for the extraction of total RNA, as suggested by our earlier protocol [29, 30]. NanoDrop 2000 (Thermo Fisher Scientific, Waltham, USA) was employed for evaluating the purity as well as the concentration of the RNA. Next, cDNA was synthesized from RNA (2 *μ*g) by RevertAid RT Reverse Transcription Kit (Thermo Fisher Scientific, Waltham). q-PCR was conducted with FastStart Universal SYBR Green master mix (Roche, Mannheim, Germany) via StepOnePlus Real-Time PCR system (Applied Biosystems, Foster City, USA).  Table 1 shows gene-specific primers for q-PCR. The reference gene *β*-actin was used as an internal control. Each sample was run in triplicate, and the 2^-*ΔΔ*Ct^ method was employed for evaluating relative mRNA expression of the target gene.

### 2.5. Western Blot Analysis

Total Protein Extraction Kit was used for the extraction of total proteins of the ileum and colon, followed by determining the concentration of proteins by Standard BCA Protein Assay Kit. The underlined kits were procured from Keygen Biotech (Nanjing, China). Western blotting was used for evaluating the expression level of proteins [29, 30]. Briefly, the separation of proteins (in equal amounts) was carried out by an SDS-PAGE, followed by transferring into a PVDF membrane (Millipore, USA). Then, skimmed milk (5%) was used for membrane blockage for 60 min at ~25°C and then overnight incubated with appropriate primary antibodies for iNOS (1 : 1000, Proteintech, China), FXR (1 : 4000, Abcam, USA), TGR5 (1 : 4000, Abcam, USA), and *β*-actin (1 : 5000, Abcam, USA) at 4°C. After washing with TBST, membranes were incubated with secondary antibodies (1 : 5000, EarthOx, USA) for 1 h at room temperature. Chemiluminescence detection was performed using an ECL luminescence reagent (Biosharp, Hangzhou, China) according to the manufacturer's instructions. Specific bands were detected, analyzed, and quantified by ImageJ software (NIH, Bethesda, MD, USA).

### 2.6. Microbiota Analysis Based on 16S rRNA High-Throughput Sequencing

Genomic DNA of cecal contents was extracted using a DNA kit (TIANGEN Biotech Co. Ltd., Beijing, China) according to the manufacturer's instructions. The NanoDrop 2000 was used for evaluating the concentration as well as purity of the extracted genomic DNA. The integrity of genomic DNA was determined by electrophoresis on 1% (*w*/*v*) agarose gels. Then, the DNA samples were sent to Majorbio Bio-pharm Technology (Shanghai, China) to perform amplicon pyrosequencing on the Illumina MiSeq platform. The distinct V3-V4 regions of the 16S rRNA genes were amplified by PCR with specific primers 338F (5′-ACTCCTACGGGAGGCAGCAG-3′) and 806R (5′-GGACTACHVGGGTWTCTAAT-3′). The clean sequences were assigned to the same operational taxonomic units (OTUs) with ≥97% similarity. The data was analyzed on the free online platform of Majorbio Cloud Platform (https://cloud.majorbio.com/). Alpha diversity, including Shannon, Simpson, Sobs, Ace, Chao1, and Coverage, was calculated to reflect the bacterial diversity and richness. Beta diversity on unweighted UniFrac was calculated based on OTU level. UniFrac-based principal component analysis (PCA) and principal coordinate analysis (PCoA) were performed to get principal coordinates and visualized from complex data. Dissimilarity in community structure between samples was calculated by nonmetric dimensional scaling (NMDS). The relative abundance of microbiota was examined at different taxonomic levels. The relative abundance of significant differences in family, genus, species, and OTU levels was calculated by the Wilcoxon rank sum test.

### 2.7. Statistical Analysis

The obtained results were represented as the mean ± SEM. GraphPad Prism version 8.0 (San Diego, USA) was used for statistical analysis. Statistical significance was assessed by a two-tailed Student's *t*-test or Wilcoxon rank sum test. *P* < 0.05 was considered statistically considerable.

## 3. Results

### 3.1. Intestinal Inflammation in Piglets after LPS Stimulation

**Figure 1:**
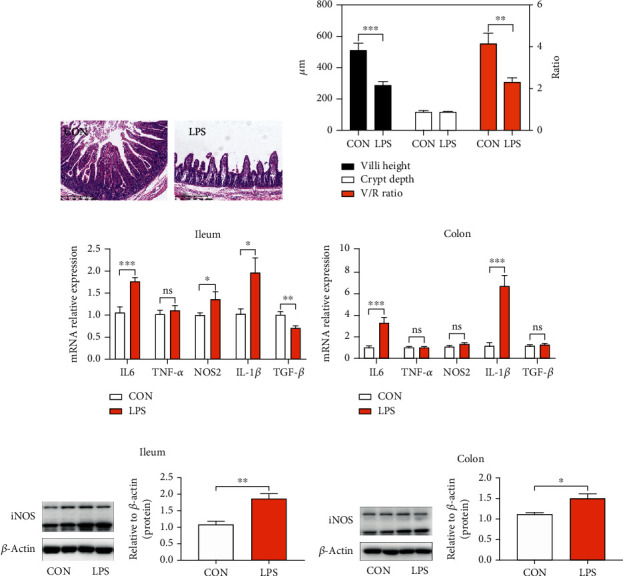
Intestinal inflammation occurred in piglets after LPS stimulation. (a) Representative H&E staining images of the distal ileum. (b) Villi height; crypt depth; the ratio of villi height : crypt depth (VCR). (c) q-PCR results of cytokine expression in the ileum. Expression was normalized to *β*-actin. (d) q-PCR results of cytokine expression in the colon. Expression was normalized to *β*-actin. (e) Immunoblot analysis of total protein extracts from piglets' ileum tissue samples. (f) Immunoblot analysis of total protein extracts from piglets' colon tissue samples. iNOS: inducible nitric oxide synthase. ^∗^*P* < 0.05, ^∗∗^*P* < 0.01, ^∗∗∗^*P* < 0.001, and ^∗∗∗∗^*P* < 0.0001 were regarded as statistically significant.

LPS is the endotoxin portion of the Gram-negative bacterial cell wall [31] and is the most abundant proinflammatory stimulus in the gastrointestinal tract. In the current study, LPS was used to induce immune responses in piglets. As shown in Figures 1(a) and 1(b), LPS stimulation considerably decreased villi height and the ratio of villi height : crypt depth (VCR) of the ileum, which showed elevated histological destruction of the ileum (caused by LPS). For further analysis, mRNA expressions of proinflammatory cytokines in the ileum and colon were also measured. The obtained results revealed that the LPS challenge considerably elevated the expression of IL-6, NOS2, and IL-1*β*, and lowered the expression level of anti-inflammatory cytokines such as TGF-*β* in the ileum, as depicted in Figure 1(c). Furthermore, LPS enhanced the expression of IL-1*β* and IL-6 in the colon, as shown in Figure 1(d). To verify the occurrence of intestinal inflammation, the protein level of proinflammatory inducible nitric oxide synthase (iNOS), the enzyme responsible for nitric oxide (NO) production, was also detected and the obtained results revealed that the expression of iNOS in the LPS group was considerably elevated in the ileum and colon, as shown in Figures 1(e) and 1(f). Taken together, LPS stimulation successfully induced intestinal inflammation and harmed the gut immunity of piglets.

### 3.2. The Variations of Gut Microbiota between Noninflammatory and Inflammatory Piglets

**Figure 2:**
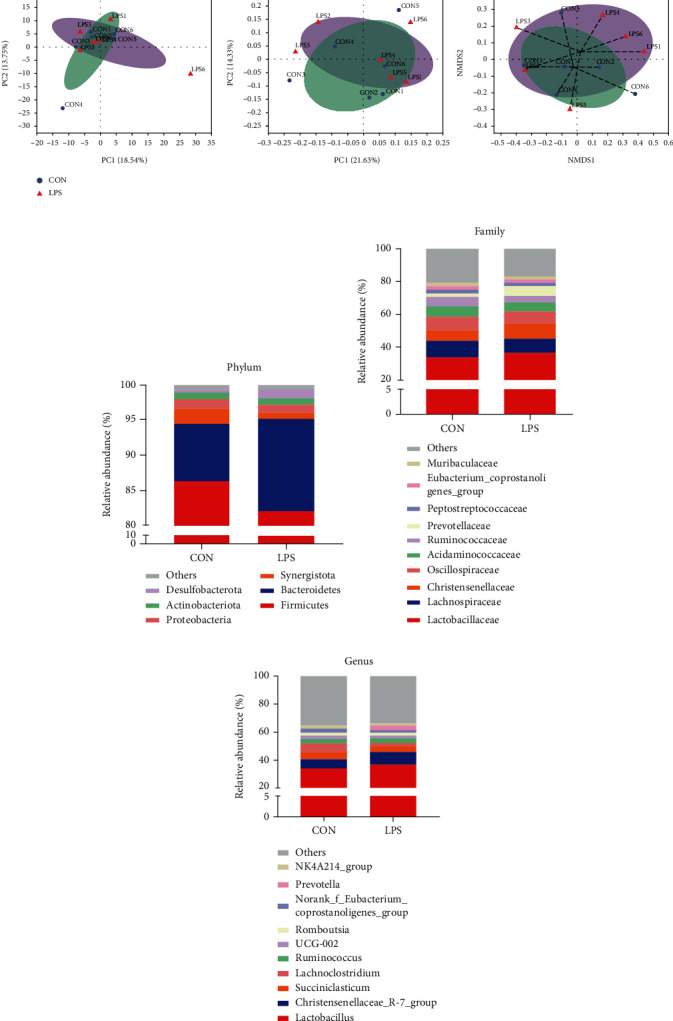
LPS changed the composition and structure of piglet's gut microbiota in the cecal contents. (a) The microbial beta diversity was accessed by principal component analysis (PCA), principal coordinate analysis (PCoA), and nonmetric multidimensional scaling (NMDS) analysis based on the OTU table. (b) Relative abundance > 1% of bacterial phyla. (c) Relative abundance of the top 10 families. (d) Relative abundance of the top 10 genera.

It has been reported in the literature that intestinal inflammation is usually accompanied by microbiota dysbiosis [32–34]. The above results showed that LPS resulted in severe inflammatory responses in the gastrointestinal tract. Whether it damages the balance of gut microbiota is unclear. Therefore, it is important to identify the differences that exist in the gut microbial richness and populations between different subject groups. 16S sequencing of cecal contents was profiled to evaluate the dynamic changes in gut microbiota. Alpha diversity index is an important indicator of species diversity and richness. Among which the coverage index, a marker of sequencing depth, indicated that the data met the requirements of subsequent analysis (Table 2). Moreover, results in Table 2 demonstrated that the species diversity and richness were not affected as accessed by Shannon, Simpson, Sobs, Ace, and Chao1 index between the CON and LPS groups. Furthermore, the structure of microbial composition was analyzed. Among various methods, the principal component analysis (PCA) and the principal coordinate analysis (PCoA) are predominant. PCA and PCoA based on unweighted UniFrac metrics showed partially distinct clustering of intestinal microbe communities of each group (Figure 2(a)). In addition, nonmetric multidimensional scaling (NMDS) ordination performed on the Bray-Curtis dissimilarity also showed that the bacterial community profiles from LPS-treated piglets were partly separated from those of samples in the CON group (Figure 2(a)). The underlined results indicated that LPS treatment leads to the change of gut microbiota structure.

The relative abundance of bacteria was further evaluated at various levels; a detailed phylogenetic analysis of the taxonomic composition of the microbiome based on OTU was conducted. At the phylum level, a total of 14 phyla were shared by piglets from all groups, and six bacteria had relative abundance exceeding 1% in at least one sample: *Firmicutes*, *Bacteroidetes*, *Synergistota*, *Proteobacteria*, *Actinobacteriota*, and *Desulfobacterota*. As shown in Figure 2(b), the major phyla in all piglets were *Firmicutes* and *Bacteroidetes*; these findings are in good agreement with other reported studies [35]. In detail, the relative abundance of *Firmicutes* (86.31%) in the CON group was slightly decreased to *Firmicutes* (82.06%) in the LPS group, while the relative abundance of *Bacteroidetes* (8.24%) in the CON group was slightly increased to *Bacteroidetes* (13.14%) in the LPS group, resulting in a lower Firmicutes-Bacteroidetes ratio. Surprisingly, there were no significant differences in the top 10 at family and genus levels between the CON group and the LPS group (Figures 2(c) and 2(d)).

**Figure 3:**
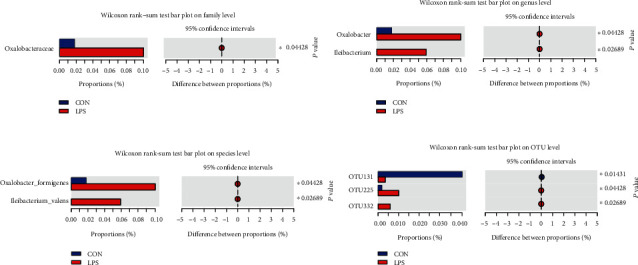
Effects of LPS challenge on the relative abundance of significant differential bacteria on (a) family, (b) genus, (c) species, and (d) OTU levels. Statistical differences between two groups were calculated by the Wilcoxon rank sum test. ^∗^*P* < 0.05 was regarded as statistically significant.

Nonetheless, variations in the microbial composition of both groups were further explored. Statistical figures revealed the relative abundance of significant differences on family, genus, species, and OTU levels. As shown in Figure 3(a), only *Oxalobacteraceae*, a family in the subclass of *Betaproteobacteria*, was upregulated by the LPS challenge. Among all genera, *Oxalobacter* and *Ileibacterium* were significantly expanded in LPS-treated piglets (Figure 3(b)). Concretely, the relative abundance of *Oxalobacter formigenes* and *Ileibacterium valens* at the species level was upregulated in the LPS group (Figure 3(c)). The Wilcoxon rank sum test based on OTU level revealed that OTU131 (*Rikenellaceae RC9 gut group*) was dramatically downregulated, while OTU225 (*Oxalobacter formigenes*) and OTU332 (*Ileibacterium valens*) were increased by LPS stimulation (Figure 3(d)). The above results indicated that LPS treatment resulted in a reshuffling of the microbiota communities.

### 3.3. Microbiota Dysbiosis Results in the Disturbance of Bile Acid Enterohepatic Circulation

**Figure 4:**
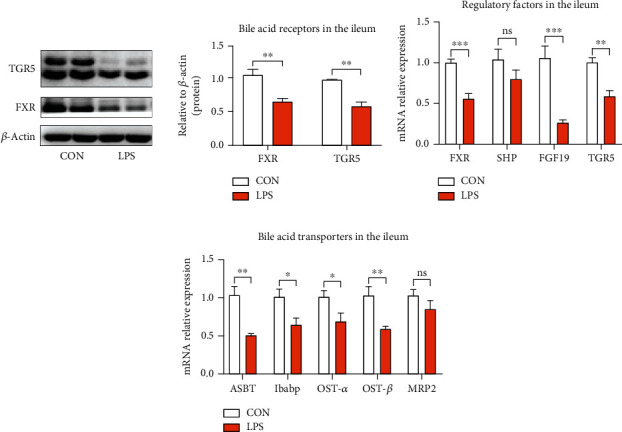
LPS damaged the ileal genes and protein expression related to enterohepatic circulation of bile acids. (a) Protein expression of bile acid receptors FXR and TGR5. FXR: farnesoid X receptor; TGR5: G protein-coupled bile acid receptor 1. (b) Expression of genes that regulate bile acid metabolism. SHP: small heterodimer partner; FGF19: fibroblast growth factor 19. (c) Expression of genes required for bile acid transportation. ASBT: apical sodium-dependent BA transporter; Ibabp: ileal bile acid-binding protein; OST-*α*: organic solute transporter subunit *α*; OST-*β*: organic solute transporter subunit *β*; MRP2: multidrug resistance-associated protein 2. ^∗^*P* < 0.05, ^∗∗^*P* < 0.01, and ^∗∗∗^*P* < 0.001 were regarded as statistically significant.

The gut microbiota regulates host immunity and metabolism through abundant microbial metabolites [4, 9, 11, 36], among which SCFAs and bile acids are the most common metabolites [17]. To the best of our knowledge, bile acids have been synthesized from cholesterol in the liver [21, 25, 37], then further metabolized by the gut microbiota, and moved through enterohepatic circulation [24, 38]. Thus, proteins associated with enterohepatic circulation were evaluated for the measurement of the underlined process. As depicted in Figure 4(a), LPS stimulation considerably attenuated the protein expression of two important BA receptors, as farnesoid X receptor (FXR) and G protein-coupled bile acid receptor 1 (GPBAR1, also called TGR5). Meanwhile, mRNA expression of the underlined receptors further validated the harmful effect of LPS on the metabolism of bile acid, as depicted in Figure 4(a).

In addition, bile acids activate FXR in enterocytes to induce the expression level of its downstream targets, *i.e.*, small heterodimer partner (SHP) and fibroblast growth factor 19 (FGF19, swine FGF19, and mouse FGF15 are homologous). The obtained results revealed a low expression level of TGR, FXR, and FGF19 in the piglets exposed to LPS, as shown in Figure 4(b). However, the expression of SHP was not affected. The underlined results suggested that LPS stimulation reduced the concentration of BAs in the ileum. Furthermore, BA-binding proteins and transporters in the ileum, such as ASBT, OST-*α*, OST-*β*, and Ibabp, were significantly inhibited by LPS, while the obvious variations were not observed in MRP2, as depicted in Figure 4(c), which results in decreasing of BAs transferring across the enterocyte for entering into the portal vein, thus disrupting enterohepatic circulation and further inducing BA de novo synthesis.

**Figure 5:**
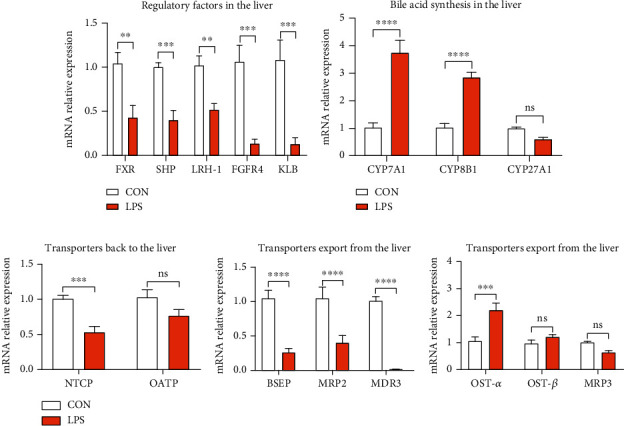
Hepatic gene expression related to enterohepatic circulation of bile acids by q-PCR. (a) Hepatic expression of genes that regulate bile acid metabolism. LRH-1: liver receptor homologue 1; FGFR4: fibroblast growth factor receptor 4; KLB: Klotho beta. (b) Hepatic expression of genes required for primary bile acid synthesis. CYP7A1: 7a-hydroxylase; CYP8B1: 12a-hydroxylase; CYP27A1: sterol 27-hydroxylase. (c) Expression of genes required for bile acid transportation. NTCP: Na^+^-taurocholate cotransporting polypeptide; OATP: organic anion transporting polypeptide; BSEP: bile salt export protein; MDR3: ATP binding cassette subfamily B member 4; MRP3: multidrug resistance-associated protein 3. ^∗^*P* < 0.05, ^∗∗^*P* < 0.01, ^∗∗∗^*P* < 0.001, and ^∗∗∗∗^*P* < 0.0001 were regarded as statistically significant.

To check the variation of bile acids in the liver, genes associated with the synthesis and transportation of BA were also evaluated. The obtained results revealed that LPS administration considerably decreased the mRNA expression of FXR and its downstream targets, *i.e.*, SHP and liver receptor homolog-1 (LRH-1), as shown in Figure 5(a). Decreased ileal FGF19 indeed led to a lower level of FGF receptor 4 (FGFR4)/*β*-Klotho (KLB) heterodimer complex (Figure 5(a)). FXR in the ileum and liver cooperated to regulate BA de novo synthesis. CYP7A1 and CYP8B1 are the hepatic genes required for primary BA synthesis, and the obtained results revealed that CYP7A1 was upregulated by LPS while CYP8B1was not affected, as shown in Figure 5(b), which agreed with the theoretical analysis. Additionally, BA transporters were also measured in the liver. Proteins encoded by Na^+^-taurocholate cotransporting polypeptide (NTCP) and organic anion-transporting polypeptide (OATP)are transporters responsible for BA uptake at the basolateral side of the hepatocytes. Results indicated that the expression level of NTCP was suppressed by LPS, while OATP was not affected by LPS, as depicted in Figure 5(c), which further induced BA de novo synthesis. Transporters encoded by bile salt export protein (BSEP), MRP2, and ATP binding cassette subfamily B member 4 (MDR3) pump BAs into the gallbladder. Results showed that LPS notably repressed the mRNA expression of BSEP, MRP2, and MDR3 (Figure 5(c)), which leads to a reduction in the level of bile acid in the intestine. The other transporters encoded by OST-*α*, OST-*β*, and multidrug resistance-associated protein 3 (MRP3) are responsible for the export of BAs from the liver into the systemic circulation. Figure 5(c) reveals that LPS considerably elevated the mRNA expression of OST-*α*, thus driving the excretion of bile acids into the systemic circulation. Collectively, LPS-induced microbial dysbiosis damaged the enterohepatic circulation of BAs and increased the expenditure of BA synthesis.

### 3.4. Disturbance of BA Enterohepatic Circulation Is Harmful to the Normal Lipid Metabolism

**Figure 6:**
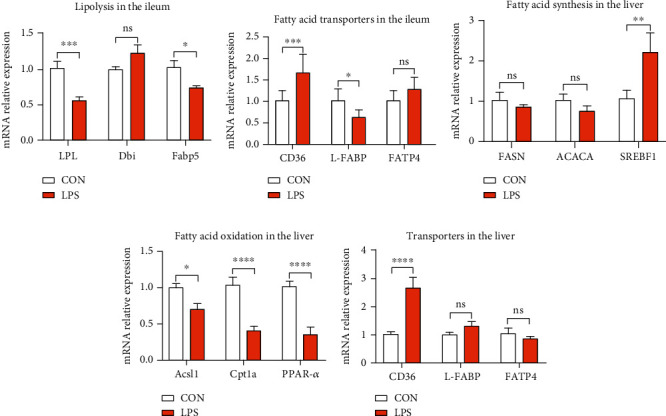
The obstruction of bile acid enterohepatic circulation made against the normal lipid metabolism. (a) Ileal expression of genes that regulate lipolysis. LPL: lipoprotein lipase; Dbi: diazepam binding inhibitor; Fabp5: fatty acid-binding protein 5. (b) Ileal gene expression of fatty acid transporters. (c) Hepatic expression of genes related to fatty acid synthesis. FASN: fatty acid synthase; ACACA: acetyl-CoA carboxylase alpha; SREBF1: sterol regulatory element binding transcription factor 1. (d) Hepatic expression of genes required for fatty acid *β*-oxidation. Acsl1: acyl-CoA synthetase long-chain family member 1; Cpt1a: carnitine palmitoyltransferase 1a; PPAR-*α*: peroxisome proliferator-activated receptor alpha. (e) Hepatic gene expression of fatty acid transporters. ^∗^*P* < 0.05, ^∗∗^*P* < 0.01, ^∗∗∗^*P* < 0.001, and ^∗∗∗∗^*P* < 0.0001 were regarded as statistically significant.

The signaling of BA in the liver and intestine contributes to the regulation of lipid metabolism [39]. For example, when stimulated, bile acids were pumped from the gallbladder into the small intestine for emulsifying and solubilizing fats for absorption [40]. The above results showed that LPS stimulation broke down the BA enterohepatic circulation. Thus, whether the disturbance of BA enterohepatic circulation had negatively affected the lipid metabolism needs further investigation. Firstly, genes related to lipolysis were measured in mRNA level. As shown in Figure 6(a), piglets challenged with LPS expressed lower lipoprotein lipase (LPL) and fatty acid-binding protein 5 (Fabp5), with no effect on diazepam binding inhibitor (Dbi) in the ileum. Then, fatty acid transporters in the ileum showed seemingly controversial results, and an upregulated expression of CD36 and reduced expression of liver fatty acid-binding proteins (L-FABP) were observed (Figure 6(b)). The above results indicated that LPS inhibited lipolysis and transportation in the intestine. In addition, we also measured genes related to fatty acid synthesis and oxidation in the liver. Fatty acid synthase (FASN) and acetyl-CoA carboxylase alpha (ACACA) are the rate-limiting enzymes in the process of fatty acid synthesis, while sterol regulatory element-binding transcription factor 1 (SREBF1) is an important transcription factor involved in lipid metabolism [41]. Results in Figure 6(c) showed that the expression of SREBF1 was significantly upregulated by LPS, whereas no change was observed in FASN and ACACA. Moreover, the expression of acyl-CoA synthetase long-chain family member 1 (Acsl1) and carnitine palmitoyltransferase 1A (Cpt1a), two essential rate-limiting enzymes of fatty acid *β*-oxidation, was suppressed by LPS (Figure 6(d)). Peroxisome proliferator-activated receptor alpha (PPAR-*α*) is a key nuclear transcription factor that affects the target genes involved in lipid metabolism, cell proliferation, cell differentiation, and immune responses, and the obtained results revealed the downregulation of the underlined transcription factor, as depicted in Figure 6(d). The fatty acid transporter, such as CD36, was considerably induced by LPS stimulation (Figure 6(e)). Above results elucidated that LPS challenge disturbed the enterohepatic circulation of bile acids and further destroyed the normal lipid metabolism.

### 3.5. Intestinal Inflammation Further Caused Liver Inflammation

**Figure 7:**
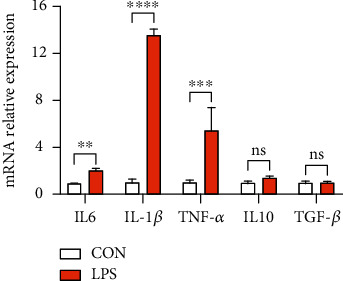
LPS stimulation triggered the gene expression of proinflammatory cytokines and further damaged liver. ^∗^*P* < 0.05, ^∗∗^*P* < 0.01, ^∗∗∗^*P* < 0.001, and ^∗∗∗∗^*P* < 0.0001 were regarded as statistically significant.

The reported studies have been revealed that intestinal inflammation can influence the liver through the gut-liver axis [42, 43]. Our results indicated that the LPS challenge contributed to severe intestinal inflammation. However, whether it can be transferred to the liver in our model is not clear. The mRNA expression of cytokines was measured. The obtained results revealed an elevated expression level of proinflammatory cytokines, *i.e.*, IL6, IL-1*β*, and TNF-*α*; however, LPS stimulation did not affect the two key anti-inflammatory cytokines, *i.e.*, IL-10 and TGF-*β*, as depicted in Figure 7. Taken together, inflammation in the gut can be transferred to the liver and may further influence host metabolism.

## 4. Discussion

Gross lesion score and histopathological examinations are commonly used for evaluating the severity of necrotic enteritis [22, 44]. The villi height, crypt depth, and VCR are important indicators for assessing intestinal function and health [45]. In this study, LPS-induced piglets showed reduced villi height and VCR in the ileum, implying the decreased surface area for nutrient absorption and harmful effect on gut health. Meanwhile, homeostasis of cytokines in the intestine also plays a pivotal role in maintaining gut immunity, including proinflammatory cytokines, *i.e.*, iNOS, IL6, IL-1*β*, and TNF-*α*, and anti-inflammatory cytokines, *i.e.*, IL-10 and TGF-*β* [46]. The reported studies revealed an elevated expression level of proinflammatory cytokines in patients affected with IBD, which implied that targeting the balance between proinflammatory and anti-inflammatory cytokines could be a potential treatment for gut inflammation [47]. In the current study, the obtained results indicated that LPS stimulation not only enhanced the mRNA expression of IL-6 and IL-1*β* but also enhanced the transcriptional and translational level of iNOS, and further induced intestinal inflammation, which showed consistency with the other reported studies [48, 49].

Some studies revealed that gut immunity has been considerably associated with gut microbiota [4, 5], and a wide range of gastrointestinal diseases, such as recurrent C. difficile infection (CDI), inflammatory bowel diseases (IBD, including Crohn's disease (CD) and ulcerative colitis (UC)), colorectal cancer (CRC), and metabolic disorders, are affected by the variations observed in the composition and functions of gut microbiota [6, 50]. Researchers implied that the variations in gut microbiota between Jinhua and Landrace pigs may contribute to the disease resistance disparity [51, 52]. Given the strict interdependence between gut immunity and the host microbiota, 16S rRNA gene sequencing of the piglets' cecum contents was conducted. Our results confirmed that LPS had no effect on microbial diversity and richness, but partially changed the structure and composition of gut microbiota, especially the upregulated abundance of *Oxalobacter formigenes* and *Ileibacterium valens*. *Oxalobacter formigenes* were first reported in 1985, which is a group of anaerobic bacteria that degrade oxalic acid and it is believed that the underlined bacteria may considerably affect the host [53]. Up to now, *Oxalobacter formigenes* has been used in a phase II study in subjects with primary hyperoxaluria type 1 and end-stage renal disease [54]. *Ileibacterium valens*, a novel member of the family *Erysipelotrichaceae*, was first reported in 2017 [55]. In light of late discovery, only one paper postulated that this bacterium might do with energy expenditure [56]. Further studies are warranted to experimentally verify the relative contribution of each enriched bacterial species and their effect on host metabolism and health.

Microbial dysbiosis results in IBD, obesity, and metabolic diseases [5, 7]. Metabolites produced by gut microbiota are considered essential intermediates between the microbiota and its host [57]. The metabolites of gut microbiota can cause modulation in the immune reactions of the host, thereby influencing their immune system and plays a considerable role in the inflammations and diseases [13]. Among them, SCFAs were the most abundant and extensively investigated metabolites [58]. In HFD mice and DSS mice, BA was evaluated as an effective metabolic factor correlated with the gut microbiota which affects the health of the host relative to SCFAs [19–21]. Our results also showed that microbial change induced by LPS stimulation is deleterious to BA enterohepatic circulation. Reduced BA regulatory factors in the ileum and liver had negative feedback on BA synthesis. As a result, increased synthesis of BA in the liver and impairment of BA uptake were also observed. The above results indicated that LPS stimulation reduced the content of BA in the gut. It has also been indicated that increased BA biosynthesis from cholesterol might prevent hepatic cholesterol accumulation, thus improving host metabolism; however, the underlined process is not clearly understood and needs further investigation.

The reported studies and accumulating evidence indicated that BA is a metabolic regulator in the intestine, which considerably affects the emulsification and absorption of fats [27, 39, 59, 60]. In this study, it has been revealed that disturbance in BA enterohepatic circulation influenced lipid metabolism. Lipolysis is the process of the breakdown of fatty acids or lipids by a chemical reaction [61]. Our results showed that reduction of BAs in the intestine prevented the expression of genes related to lipolysis and fatty acid transporters, and thus damaged lipid metabolism in the gastrointestinal tract. Furthermore, the variations were also observed in the *de novo* synthesis and oxidation of fatty acid in the liver, which leads to further deposition of fat in the liver. The underlined process can trigger lipid metabolic reprogramming. The above results indicated that BA enterohepatic circulation is closely related to host lipid metabolism. Other reported studies also have confirmed that various types of BAs exert different effects on lipid metabolism in mice and humans [37, 62, 63]. However, there is still a lack of clarity regarding the association between the metabolism of bile acids and lipid which needs further exploration.

Intestinal inflammation can be transferred to the liver through the gut-liver axis [42] and further impacts host metabolism in the liver [59]. Our results revealed an elevation in the level of proinflammatory cytokines in the liver (upon LPS stimulation), which indicated hepatic inflammation. Our observations contrasted with published data together suggested that intestinal damage induced by various stimulators enhanced hepatic inflammation [44]. Whether liver injury dampened host metabolism is vague and limited in our study.

In the current study, we evaluated the effects of gut immunity and microbial dysbiosis on bile acids and lipid metabolism in the piglets exposed to LPS. However, some areas are poorly explored and need further investigation, *i*.*e*., the bacteria that regulate the metabolism of bile acids, the association of microbiome with host metabolism, and the relationship between host immunity and gut microbiota. Besides, their potential mechanism also needs to be further explored.

## 5. Conclusion

In conclusion, the obtained results revealed that LPS stimulation results in intestinal erosion and the release of proinflammatory cytokines further lead to profound changes in the gut microbial composition and structure, especially the relative abundance of *Oxalobacter formigenes* and *Ileibacterium valens*. The underlined variations led to a disturbance in the enterohepatic circulation of BAs, which further damages lipid metabolism. However, there is still a lack of clarity regarding the relationship of gut immunity and intestinal microbiota with host metabolism and their potential mechanism which needs further investigation.

## Figures and Tables

**Table 1:** Primer sequences for q-PCR.

Gene	Primer sequence (5′ ⟶ 3′)	GenBank number
ACACA	Forward: TGGACAGGGCTCTTACCTGT Reverse: GAGACCAGTGAAGGCTGCTT	XM_021066229.1
Acsl1	Forward: GTCCTTCCTCCGATGATACTCTG Reverse: GGACCACAGGGAAGATGGTG	NM_001167629.2
ASBT	Forward: CCAGAGTGCCTGGATCATCG Reverse: GGAGTAACCGGCCAAAGGAA	NM_001244463.1
*β*-Actin	Forward: GAAGCTGTGATGGACGCAGG Reverse: CCTGGAGAGGTTCACCGGAA	XM_021086047.1
BSEP	Forward: CGCAGCGTGAAGAAATGTGG Reverse: AACCGAAACAGTTGAAAGAGGC	XM_003133457.5
CD36	Forward: TAGGAATCCCACTGCCTCAC Reverse: GCTTCAAGTGCTGGGTCAAA	NM_001044622.1
CPT1A	Forward: TGGTGTCCAAATACCTCGCC Reverse: GATAATCGCCACGGCTCAGA	NM_001129805.1
CYP27A1	Forward: GAGGGCAAGTACCCAGTACG Reverse: TGACTCTCCTTCCGTGGTGA	NM_001243304.1
CYP7A1	Forward: CCGCTTCTGATACCTGTGGA Reverse: GGTTTGCTCGGAGGAACTCA	NM_001005352.3
CYP8B1	Forward: CAAGTTCGACCGCATGTTCC Reverse: TTATGCCGTGCCTCTCCAAG	NM_214426.1
Dbi	Forward: GCCACTACAAACAAGCGACC Reverse: TTGGAAGTCCCTTTCAGCCC	NM_214119.1
FABP5	Forward: AGGCACCAGTCCGCTTATTC Reverse: TTTCGTAGGGCCATTCCCAC	NM_001039746.2
FASN	Forward: CGTTGGGTCGACTCACTGAA Reverse: GAGACAGTTCACCATGCCCA	NM_001099930.1
FATP4	Forward: CAGAGTGGCTGTCGTTCCG Reverse: GGTTACCAACCTCCCAGCAAG	XM_021069619.1
FGF19	Forward: TGAGTACCGTGGCGATCAAG Reverse: GCGGATCTCCTCCTCGAAAG	XM_003122420.3
FGFR4	Forward: CCAGGAGTTCTTTGCCTTCTC Reverse: GCGACTACCCTCCTTGTACC	XM_013987555.2
FXR	Forward: TGAGCTTTGTGTCGTTTGCG Reverse: ACATTCAGCCAACATTCCCATC	NM_001287412.1
IBABP	Forward: GCGACATAGAGACCATCGGG Reverse: GTAGTTGGGGCTGTTCACCA	NM_214215.2
IL-10	Forward: GGGTGTGCCCTATGGTGTTC Reverse: GGGTGGGTAGGCTTGGAATG	NM_214041.1
IL-1*β*	Forward: CCAGCCAGTCTTCATTGTTCA Reverse: GCTGGATGCTCCCATTTCTC	NM_214055.1
IL-6	Forward: ACAAAGCCACCACCCCTAAC Reverse: CGTGGACGGCATCAATCTCA	NM_214399.1
KLB	Forward: ATCGACGACCAGTCTCTGGA Reverse: TGACTTTATCAAGCAGGTGTGC	XM_003482367.4
L-FABP	Forward: CATCACTACCGGGTCCAAGG Reverse: TTCTCCCCAGTCAGGGTCTC	NM_001004046.2
LPL	Forward: CAGCCCTGGCTTTGCTATTGA Reverse: GACTCCACGTGCTGTTCCTTC	NM_214286.1
LRH-1	Forward: CGAAGAGCTCTGTCCTTACTGTC Reverse: GTCCATTGGCTCGGATGAGG	NM_001267893.1
MDR3	Forward: AAACCGGGTGTCCTCAGACT Reverse: TCGGGGAGATCGACCAGATT	XM_021063468.1
MRP2	Forward: GGCTACTCCTGCGTGTTCTT Reverse: TCCTCAGCAACATCCCACAC	XM_021073710.1
MRP3	Forward: GGTTGGAAGGCCACCGTTTT Reverse: GTGTGCAAGGACAGGTTGGA	XM_003131575.6
NOS2	Forward: TTGAATCTGGGTGAAGAGCCC Reverse: GCGGTGAAGTGTGTCTTGGA	NM_001143690.1
NTCP	Forward: TTCCCTGCACCATAGGCATC Reverse: CGAGCATTGAGGCGGAAAAG	XM_001927695.5
OATP	Forward: CAGAAGATCCATCAGAGTGTGTGA Reverse: GTGTTCACCGATCCAGTGTCA	XM_021091164.1
OST-*α*	Forward: GACGGAGCCAGAAGGAAAGAC Reverse: CAGACGGAGGGGATGCTGTA	NM_001244266.1
OST-*β*	Forward: GGCGTGTGCTAAATGCAGAG Reverse: GTTTTCCACACGGCTGTCAC	XM_005658570.3
PPAR-*α*	Forward: GAAGTACGGCGTCTACGAGG Reverse: CGCACCAAATGATAGCAGCC	NM_001044526.1
SHP	Forward: TGCTGCCTGGAGTCCTTATG Reverse: ACAGGGCGAAAGAAGAGGTC	XM_003127720.4
SREBF1	Forward: GAGCCGCCCTTCACAGAG Reverse: GTCTTCGATGTCGGTCAGCA	NM_214157.1
TGF-*β*	Forward: GAGAGCCTCAACTTCCCTCC Reverse: CCATGTCGATGGTCTTGCAG	NM_214015.2
TGR5	Forward: AGATTAGCTGAGCGGTAGCAGG Reverse: CCATGGCTTGCCATCAAGGT	XM_013984487.2
TNF-*α*	Forward: CGACTCAGTGCCGAGATCAA Reverse: CTCACAGGGCAATGATCCCA	NM_214022.1

**Table 2:** The microbial alpha diversity based on whole OTU table in the cecal contents of piglets challenged with LPS.

Items	CON	LPS	*P* value
Shannon	3.2765 ± 0.3300	3.1117 ± 0.6580	>0.9999
Simpson	0.0949 ± 0.0284	0.1418 ± 0.1081	>0.9999
Sobs	250.2000 ± 29.9370	244.8000 ± 27.7070	0.9975
Ace	303.3700 ± 34.3760	296.9600 ± 15.9010	0.9937
Chao1	304.3800 ± 34.2980	304.2200 ± 16.3850	>0.9999
Coverage	0.9980 ± 0.0002	0.9980 ± 0.0002	>0.9999

Data is presented as the mean ± SEM (*n* = 6). All numbers were unified as 0.0000. *P* values are from the Wilcoxon rank sum test.

## Data Availability

The datasets used and/or analyzed during the current study are available from the corresponding author on reasonable request.
